# Quality of Life of Patients With Nasal Obstruction Who Underwent Septoplasty: Assessed With the Glasgow Benefit Inventory

**DOI:** 10.7759/cureus.45523

**Published:** 2023-09-19

**Authors:** Feras Alkholaiwi

**Affiliations:** 1 Department of Otorhinolaryngology-Head and Neck Surgery, College of Medicine, Imam Mohammad Ibn Saud Islamic University (IMSIU), Riyadh, SAU

**Keywords:** nose, outcome, septoplasty, quality of life, glasgow benefit inventory

## Abstract

Background: Septoplasty is considered the definitive treatment for symptomatic patients with deviated nasal septum. Although septoplasty is a commonly successful performed surgery, it has also been found to affect patients’ quality of life (QoL). The Glasgow Benefit Inventory (GBI) scale is a validated questionnaire used to assess satisfaction after treatment.

Objective: The present study was conducted with the aim to assess the outcome of septoplasty using the GBI scale.

Methods: A retrospective study was conducted among patients with chronic nasal obstruction in Dr. Sulaiman Alhabib Hospital, Riyadh, Saudi Arabia, to assess the outcome of septoplasty using the GBI. All patients who met the eligibility criteria were subjected to a detailed otorhinolaryngology, head and neck examination, including nasal endoscopy prior to septoplasty, followed by the distribution of a previously validated questionnaire translated into Arabic. Statistical analysis was performed using R version 3.6.3 software. Counts and percentages were used to summarize the distribution of categorical variables.

Results: A total of 75 patients were enrolled in the study initially, among which 42 patients met the eligibility criteria; 65% were male and 35% were female. Most respondents reported improvement in the total GBI score (92.5%, *n* = 40). A similar improvement was observed in the general subscale domain (92.5%, *n* = 40) as compared to the social support domain (66.7%, *n* = 28) and physical health domain (66.7%, *n* = 28), which showed less improvement.

Conclusion: We found a positive change in the QoL among the patients after septoplasty using a validated and reliable instrument.

## Introduction

Nasal obstruction is considered to be the most common complaint in rhinological practice caused by a variety of etiological factors, of which the contributing factor is nasal septal deviation [1]. Deviated nasal septum is caused by abnormalities of bony and cartilaginous structures of the septum that can lead to difficulties in breathing caused by reduced nasal airﬂow. According to a survey, approximately 80% of individuals have a deviated nasal septum, of which only a few have severe deviation causing a nasal obstruction [2]. The prevalence of nasal septal deviation ranges from 19% to 65% [3]. However, in Saudi Arabia, a recent study determined a prevalence rate of 75.2% having a septal angle is 5° or more [4]. Septoplasty is considered the most appropriate treatment for deviated nasal septum as it improves nasal airflow and resolves symptom due to nasal obstruction [5-7]. This procedure is considered the third most common in the field of otorhinolaryngology, head and neck surgeries [8].

The quality of life (QoL) has been considered an important assessment in clinical interventions as it is used to assess the effects of a disease and the therapeutic effects of its treatment on the patient’s health. Nowadays, the QoL is becoming a criterion for the success of medical treatments as it not only includes the symptoms of the disease but also the daily life activities related to social, physical, and emotional problems. Overall, it is concerned with the general feeling of the patient either after a disease or treatment [9]. The outcome measures help to achieve the most effective treatment in a healthcare-oriented setting. Although septoplasty is a commonly successful surgery, it has also been found to affect patients’ QoL. The subjective assessment of the QoL can be done by various scales, such as Derriford Appearance Scale-Modified 24 (DAS-Modified 24) to rate the preoperative appearance-related distress [10], Rhinoplasty Outcomes Evaluation (ROE) used for preoperative and postoperative aesthetic evaluation, Nasal Obstruction Symptom Evaluation (NOSE) for functional evaluation [11-13], and Glasgow Benefit Inventory (GBI) to assess postoperative benefit after surgery [14]. Despite the fact that the scales are subjective in nature, they are considered a vital element toward the success of the treatment. Several studies have investigated the effect of septoplasty on patients' QoL using these scales. A prospective study done by Stewart et al. among 59 septoplasty patients found significant improvement in the nasal obstruction septoplasty effectiveness (NOSE) scales at three months post surgery [13]. Simsek et al. found a statistically significant correlation between the scores of postoperative GBI scores among patients with higher preoperative DAS-24 and NOSE scores [15]. Another study showed improved QoL on 50 patients with nasal obstruction using the GBI and NOSE scales [16].

Although the effectiveness of the septoplasty procedure was proved by various scales, the scales used in most of these studies do not cover all required aspects of the QoL and do not assess the patients postoperatively. In the present study, we have used the GBI scale as it is a validated questionnaire to assess the satisfaction of patients post intervention and the benefit of otorhinolaryngological procedures. It includes various questions related to several aspects of QoL and their answers on a ﬁve-point Likert scale. The scores range from -100 (maximal deterioration) through 0 (no change) to +100 (maximal improvement) [17,18]. Our study was done to assess the outcomes of septoplasty using the Arabic version of the previously validated GBI questionnaire [19].

## Materials and methods

A retrospective study was conducted among patients with deviated nasal septum who underwent septoplasty at Dr. Sulaiman Alhabib Hospital, Riyadh, Saudi Arabia, from March 2021 to June 2022, to assess the outcome using the GBI. 

Patients aged 18 years and above presenting with complaints of chronic nasal obstruction (lasting for at least six months) and deviated nasal septum and underwent septoplasty were included in the study. The patients with a history of previous nasal surgery, sinonasal malignancy, history or evidence of chronic sinusitis, allergic rhinitis, radiation therapy to the head and neck, recent nasal trauma or fracture in the past three months, septal perforation, craniofacial syndrome, nasal valve collapse, nasopharyngeal mass, adenoid hypertrophy, Wegener granulomatosis, and sarcoidosis and those who had undergone other ENT procedures, such as rhinoplasty and sinus surgery concomitant to septal surgery, were excluded from the study.

All patients who met the eligibility criteria and agreed to participate by giving signed informed consent were enrolled in the study. The participants were subjected to a detailed otorhinolaryngology, head and neck examination, including nasal endoscopy prior to septoplasty. A previously published questionnaire related to the GBI was translated and validated into the Arabic language [19].

The GBI was used to assess the quality of life [14,17,18,19]. It includes an overall score in addition to the scores for three subscales: general subscale (remaining score if questions of social support and physical health are excluded), social support subscale, and physical health subscale. Each question is scored on a five-point Likert scale, where 1 refers to a large deterioration in health status and 5 refers to a large improvement in health status. The scores are then averaged and transformed into a beneﬁt scale ranging from -100 (maximal negative beneﬁt), 0 (no beneﬁt), to +100 (maximal positive beneﬁt).

These scores are obtained as follows: 1. general score: 1) Adding scores of questions 1 to 18, 2) dividing by 18, 3) subtracting 3 from the result, and 4) multiplying by 50; 2. general subscale: 1) adding scores of questions 1 to 6, 9, 10, 14, 16 to 18, 2) dividing by 12, 3) subtracting 3 from the result, and 4) multiplying by 50; 3. social support subscale: 1) adding questions 7, 11, and 15, 2) dividing by 3, 3) subtracting 3, and 4) multiplying by 50; 4. physical health subscale: 1) adding questions 8, 12, and 13, 2) dividing by 3, 3) subtracting 3 from the result, and 4) multiplying by 50.

Ethical approval

The Institutional Review Board of College of Medicine at Imam Mohammad Ibn Saud Islamic University (IMSIU, Riyadh, Saudi Arabia) issued approval (approval no. 287/2022), dated February 22, 2022. Ethical approval and informed consent were obtained from the patients prior to filling the GBI questionnaire. The study was conducted in accordance with the Declaration of Helsinki. 

Statistical analysis

Statistical analysis was performed using R version 3.6.3. Counts and percentages were used to summarize the distribution of categorical variables. Hypothesis testing was performed at a 5% level of significance.

## Results

A total of 75 patients were initially included; only 42 patients who met the inclusion criteria and underwent septoplasty were enrolled in the study. Males and females represented 65% and 35% of the study sample, respectively. Most of the respondents were Saudis (92.86%). Time since operation varied from three months (42.9%) to one year (26.2%) (Table 1).

**Table 1:** Descriptive statistics for the study sample

	[ALL]	N
	N= 42	
Gender:		20
Female	15 (20.0%)	
Male	27 (65.0%)	
Age	31.4 (7.32)	42
Nationality:		42
Non-Saudis	3 (7.14%)	
Saudis	39 (92.86%)	
Time since operation:		42
3 months	18 (42.9%)	
6 months	6 (14.3%)	
9 months	7 (16.7%)	
12 months	11 (26.2%)	

Table 2 indicates an improved quality of life across all domains. Nonetheless, 50% of the patients thought that the operation did not affect their embarrassment or self-confidence, and two-thirds thought that the operation did not affect their tendency to avoid social situations, nor did they think it affected the number of people who cared about them. In nine of the questions, the percentages of neutral respondents ranged from 42% to 69%. The percentage of neutral respondents was less than 10% for four questions. The percentage of respondents who reported an improvement in the QoL was >50% in eight questions.

**Table 2:** Responses to individual GBI items Higher scores indicate better quality of life, with (3) indicating no change. GBI: Glasgow Benefit Inventory

	1	2	3	4	5
Has the result of the operation/intervention affected the things you do?	0 (0.00 %)	1 (2.38 %)	3 (7.14 %)	6 (14.29 %)	32 (76.19 %)
Have the results of the operation/intervention made your overall life better or worse?	0 (0.00 %)	2 (4.76 %)	1 (2.38 %)	8 (19.05 %)	31 (73.81 %)
Since your operation/intervention, have you felt more or less optimistic about the future?	0 (0.00 %)	2 (4.76 %)	11 (26.19 %)	13 (30.95 %)	16 (38.10 %)
Since your operation/intervention, do you feel more or less embarrassed when with a group of people?	1 (2.38 %)	1 (2.38 %)	21 (50.00 %)	6 (14.29 %)	13 (30.95 %)
Since your operation/intervention, do you have more or less self-confidence?	0 (0.00 %)	0 (0.00 %)	19 (45.24 %)	13 (30.95 %)	10 (23.81 %)
Since your operation/intervention, have you found it easier or harder to deal with the company?	0 (0.00 %)	0 (0.00 %)	19 (45.24 %)	11 (26.19 %)	12 (28.57 %)
Since your operation/intervention, do you feel that you have more or less support from your friends?	0 (0.00 %)	0 (0.00 %)	20 (47.62 %)	12 (28.57 %)	10 (23.81 %)
Have you been to your family doctor, for any reason, more or less often, since your operation/intervention?	1 (2.38 %)	2 (4.76 %)	22 (52.38 %)	7 (16.67 %)	10 (23.81 %)
Since your operation/intervention, do you feel more or less confident about job opportunities	0 (0.00 %)	0 (0.00 %)	25 (59.52 %)	5 (11.90 %)	12 (28.57 %)
Since your operation/intervention, do you feel more or less self-conscious?	5 (11.90 %)	4 (9.52 %)	16 (38.10 %)	11 (26.19 %)	6 (14.29 %)
Since your operation/intervention, are there more or fewer people who really care about you?	1 (2.38 %)	1 (2.38 %)	29 (69.05 %)	4 (9.52 %)	7 (16.67 %)
Since you had the operation/intervention, do you catch colds or infections more or less often?	3 (7.14 %)	1 (2.38 %)	12 (28.57 %)	10 (23.81 %)	16 (38.10 %)
Have you had to take more or less medicine for any reason since your operation/intervention?	2 (4.76 %)	3 (7.14 %)	18 (42.86 %)	8 (19.05 %)	11 (26.19 %)
Since your operation/intervention, do you feel better or worse about yourself?	1 (2.38 %)	2 (4.76 %)	2 (4.76 %)	21 (50.00 %)	16 (38.10 %)
Since your operation/intervention, do you feel that you have you had more or less support from your family?	0 (0.00 %)	0 (0.00 %)	16 (38.10 %)	11 (26.19 %)	15 (35.71 %)
Since your operation/intervention, are you more or less inconvenienced by your health problem?	3 (7.14 %)	2 (4.76 %)	3 (7.14 %)	14 (33.33 %)	20 (47.62 %)
Since your operation/intervention, have you been able to participate in more or fewer social activities?	0 (0.00 %)	2 (4.76 %)	17 (40.48 %)	12 (28.57 %)	11 (26.19 %)
Since your operation/intervention, have you been more or less inclined to withdraw from social situations?	0 (0.00 %)	5 (11.90 %)	27 (64.29 %)	3 (7.14 %)	7 (16.67 %)

Most respondents reported improvement in the total GBI score (92.5%, *n* = 40). A similar improvement was observed in the general subscale domain (92.5%, *n* = 40) as compared to the social support domain (66.7%, *n* = 28) and physical health domain (66.7%, *n* = 28), which showed less improvement (Table 3).

**Table 3:** Descriptive statistics for the overall and subscales of the GBI Improvement was defined as a score > 0. GBI: Glasgow Benefit Inventory

Score	Mean	SD	median	Trimmed mean	mad	min	max	range	N (%) Improved
Total	41.20	26.83	36.11	40.77	24.71	-11.11	97.22	108.33	40 (92.5%)
General subscale	45.04	27.49	39.58	45.71	24.71	-25.00	100.00	125.00	40 (92.5%)
Social support subscale	34.92	35.65	33.33	31.86	49.42	-16.67	100.00	116.67	28 (66.7%)
Physical health subscale	32.14	43.30	33.33	33.33	49.42	-83.33	100.00	183.33	28 (66.7%)

**Figure 1:**
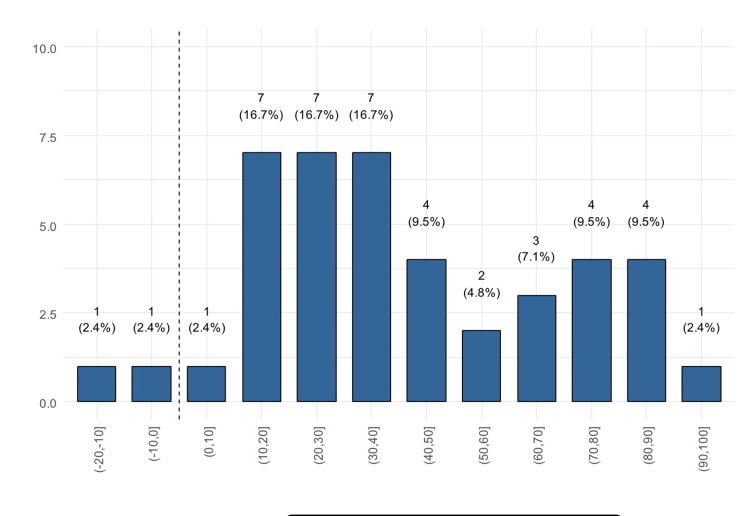
Distribution of the GBI total score (patients to the right of the dashed line reported improvement) GBI: Glasgow Benefit Inventory

**Figure 2:**
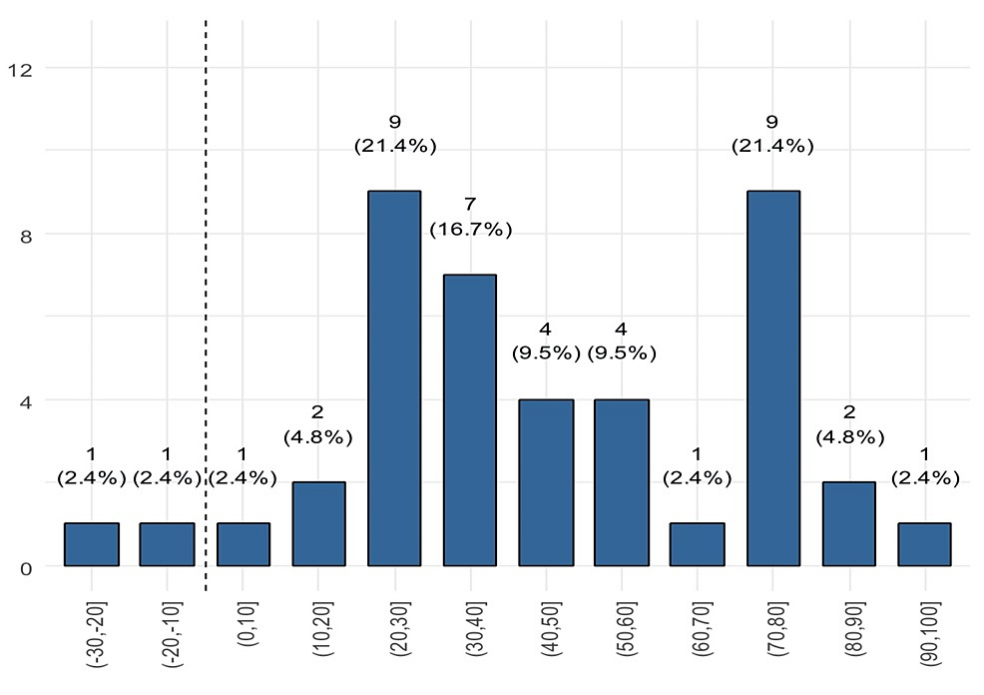
Distribution of the general subscale score (patients to the right of the dashed line reported improvement)

**Figure 3:**
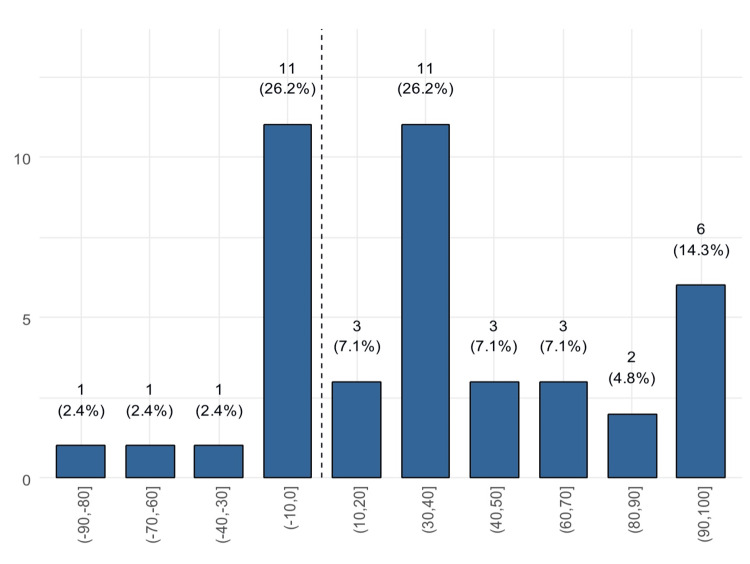
Distribution of the physical health subscale score (patients to the right of the dashed line reported improvement)

**Figure 4:**
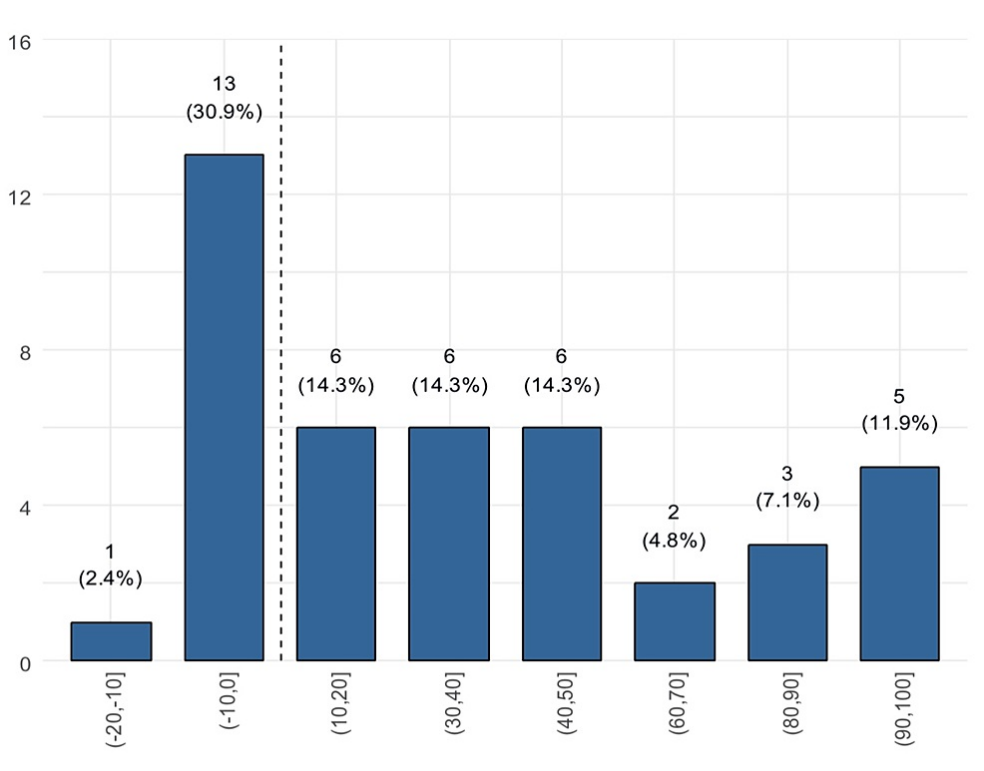
Distribution of the social support subscale score (patients to the right of the dashed line reported improvement)

We found that only two patients experienced worsening in the total GBI scores compared to 40 who reported improvement (Figure 1). Twelve patients (28.6%) reported an improvement of >70 points (Figure 2). Only 28 (66.7%) patients reported improvement on the physical health subscale (Figure 3) and also on the social support subscale, while 14 reported worsening. Of these, 30.9% reported a worsening of 1 to 10 points (Figure 4).

## Discussion

Nasal pathologies, such as deviated nasal septum, nasal polyp, turbinate hypertrophy, concha bullosa, and other sinonasal disorders, result in nasal obstruction causing a high level of psychological distress and affect patients’ QoL [20]. The septoplasty procedure is the third most frequently performed procedures by otorhinolaryngologists. Although this method is found successful by most people, assessment of the patient using a variety of scales is a must. Our study used a GBI questionnaire to assess the patients' satisfaction after the septoplasty procedure and the impact of the procedure on the patients.

One of the ways to predict the outcome of any surgical procedure regarding patient satisfaction is through QoL questionnaires. The GBI is an 18-item post-interventional questionnaire developed by Robinson et al. to measure patient benefit, particularly after otorhinolaryngological procedures along psychological, social, and physical subscales [14,18]. This scale is widely used in the otorhinolaryngology specialty as it is purposely designed by ENT scientists for ENT-related operations. It gives a total score that ranges from +100 (maximum positive change) to −100 (maximum negative change) [14]. Many previous authors, including Herruer et al. [21], Kotzampasakis et al. [22], and Chauhan et al. [23], recommended the use of the GBI to assess postoperative patient satisfaction after facial cosmetic surgeries. Owing to its high validity, reliability, and specificity [20,22], it has been translated into many languages and used in well over 100 studies [14]. We have used the Arabic version of the GBI taken from a previous study conducted by Aldriweesh et al. [19], having reliability higher than 0.70 for the total score and the three subscales using Cronbach’s alpha.

In the present study, most respondents reported improvement in the total GBI score (92.5%, *n* = 40). Similarly, Simsek et al. found that the postoperative mean was 47.73 ± 28.20 and 96.00% patients had a positive benefit while only 2.00% patients had a negative benefit [15]. In Kotzampasakis et al.'s study, a total of 92 patients (92/100) reported improvement in their QoL after the septoplasty and only eight were found to worsen. The patients’ overall QoL markedly improved, reaching a mean of 80% in the GBI [22]. In another study, Chauhan et al. found a mean total score of 53.8 using the GBI among 30 adolescents with rhinoplasty and concluded that the GBI is a reliable inventory to assess the QoL among patients with rhinoplasty [23]. Calder and Swan [24] and Valsamidis et al. [7] found a mean total GBI score of 11.3 and 19.86, respectively, after surgery. Aldriweesh et al. found that the mean beneﬁt of the GBI total score was 30.0 ± 36.37 among 51 patients undergoing otolaryngology interventions [19]. Rojas et al. [25] and Uppal et al. [26] found improved QoL among 75% and 65.3% of patients after using the GBI. Musani et al. reported a significant improvement in the general health status by using the GBI in septoplasty patients postoperatively first, third, and eighth weeks [27]. Banerjee et al. concluded that the GBI index was the subjective and patient-dependent tool used to measure the QoL of patients operated on for septal deviation [28]. Although the GBI questionnaire has high reliability and validity, a study done by Balikci et al. showed that the GBI scores were not as high as expected among patients with very good surgical outcomes [29].

In the present study, out of three domains, more improvement was observed in the general subscale domain (92.5%, *n* = 40) as compared to the social support domain (66.7%, *n* = 28) and physical health domain (66.7%, *n* = 28), whereas in Kotzampasakis et al.'s study, 97 patients reported better QoL, and only three patients reported worse QoL in the social support subscale [22]. Chauhan et al. found the social support subscale score of 32.2 and physical health subscale score of 18.3 [23]. Valsamidis et al. found scores of 22.49, 20.83, and 5.2 for the general benefit, physical health, and social support subscales, respectively [7]. Aldriweesh et al. found a score of 35.21 ± 25.98 for general health, 25.81 ± 45.98 for physical beneﬁt, and 29.08 ± 34.45 for social support among patients of Saudi Arabia [19].

Although there are numerous scales to subjectively assess the patient’s QoL after the surgery, the comparison of these scales is quite difficult. Most of the studies have found similar results between two or three scales. A study comparing the NOSE and GBI scales on 50 patients with nasal obstruction showed improved QoL scores after septorhinoplasty [16]. Simsek et al. commented that there were no significant differences between age groups with regard to surgery type, DAS-24 scores, GBI scores, and surgeon evaluation [15], whereas Rojas et al. found an inversely proportional correlation between the NOSE and GBI scales (0.3682) [25]. Schwentner et al. conducted a retrospective study including 285 patients who had undergone septorhnoplasty using the GBI and Health-Related Quality of Life Questionnaire (HRQoL), and no signiﬁcant postoperative change was reported in the subgroups of emotion, social life, and accompanying symptoms [30]. Konstantinos et al. found a statistically signiﬁcant relationship between nasal patency and mean postoperative NOSE and GBI scores as the patients with greater improvements in nasal patency and nasal obstruction symptoms (better NOSE scores) had better QoL (better GBI results) scores postoperatively [22]. Valsamidis et al. found no correlation between the mean NOSE and GBI scores and the acoustic rhinometry measurements [7].

One of the study's key strengths lies in its assessment of patients at various time intervals (ranging from three months to one year) following septoplasty in patients, coupled with the utilization of a well-validated measurement tool. However, there are certain limitations to this study, such as the relatively small sample size and the absence of alternative rating systems like the Sino Nasal Outcome Test-20 (SNOT 20) questionnaire and NOSE for comparative purposes. Hence, prospective studies enrolling a larger number of patients are necessary.

## Conclusions

Septoplasty is a common rhinological procedure to improve the QoL of symptomatic patients with deviated nasal septum. Our study found that most respondents reported improvement in the total GBI score and the general subscale among the three domains. We found a positive change in the QoL among the patients after septoplasty using a validated GBI questionnaire.
